# Comparisons between the 2012 New CKD-EPI (Chronic Kidney Disease Epidemiology Collaboration) Equations and Other Four Approved Equations

**DOI:** 10.1371/journal.pone.0084688

**Published:** 2014-01-13

**Authors:** Ying Zhu, Xiaoshuang Ye, Bei Zhu, Xiaohua Pei, Lu Wei, Jianqing Wu, Weihong Zhao

**Affiliations:** 1 Division of Nephrology, Department of Geriatrics, The First Affiliated Hospital of Nanjing Medical University, Nanjing, Jiangsu, China; 2 Department of Nephrology, Huai'an First People's Hospital, Huai'an, Jiangsu, China; 3 Division of Respiration, Department of Geriatrics, The First Affiliated Hospital of Nanjing Medical University, Nanjing, Jiangsu, China; UNIFESP Federal University of São Paulo, Brazil

## Abstract

**Objective:**

The Chronic Kidney Disease Epidemiology Collaboration (CKD-EPI) reported two equations in 2012: one based on cystatin C concentration (CKD-EPI_2012cys_) and the other using both serum creatinine and cystatin C concentrations (CKD-EPI_2012Scr-cys_). We compared the adaptability of new formulae with other four equations.

**Methods:**

Participants (n = 788; median age, 54 [range, 19–94] years) were recruited from the First Affiliated Hospital of Nanjing Medical University. The reference glomerular filtration rate (rGFR) was measured by a^ 99^mTc-DTPA renal dynamic imaging method, and the estimated glomerular filtration rate (eGFR) was calculated separately by the Chinese adapted Modification of Diet in Renal Disease equation (C-MDRD), MacIsaac, Ma, serum creatinine-based CKD-EPI equation (CKD-EPI_2009Scr_), CKD-EPI_2012cys_ and CKD-EPI_2012Scr-cys_ equations. We compared the performance of six equations with rGFR.

**Results:**

Median rGFR was 76.35 (interquartile range, 59.03–92.50) mL/min/1.73 m^2^. Compared with CKD-EPI_2009Scr_, CKD-EPI_2012Scr-cys_ formula had better diagnostic value with larger area under the receiver operating characteristic curve (ROC^AUC^, 0.879, p = 0.006), especially in young participants (ROC^AUC^, 0.883, p = 0.005). CKD-EPI_2012cys_ equation did not perform better than other available equations. Accuracy (the proportion of eGFR within 30% of rGFR [P_30_]) of the CKD-EPI_2012Scr-cys_ equation (77.03%) was inferior only to MacIsaac equation (80.2%) in the entire participants, but performed best in young participants with normal or mildly-injured GFR. Neither of the two new CKD-EPI equations achieved any ideal P_30_ in the elderly participants with moderately-severely injured GFR. Linear regression analysis demonstrated a consistent result. In this study, CKD-EPI_2012Scr-cys_ had a relatively better diagnosis consistency of GFR stage between the eGFR and rGFR in the whole cohort.

**Conclusion:**

CKD-EPI_2012Scr-cys_ appeared less biased and more accurate in overall participants. Neither of the new CKD-EPI equations achieved ideal accuracy in senior participants with moderately-severely injured GFR. A large-scale study with more subjects and cooperating centers to develop new formulae for the elderly is assumed to be necessary.

## Introduction

Chronic kidney disease (CKD) has become a serious threat to human health worldwide [1]. Increasing prevalence of diabetes, hypertension, and obesity will result in an even greater burden of CKD in developing countries such as China [2]. In *The Lancet*, Zhang et al. presented the results of the first comprehensive study exploring the prevalence of CKD in China. The prevalence was 10.8% in 2012, equivalent to 119.5 million CKD patients [3]. Outcomes of CKD include not only progression to kidney failure but also a series of complications [4]. Early impaired kidney function often has no obvious symptoms, which leads to easily missed or delayed diagnosis. Therefore, accurate assessment of kidney function is essential, which needs not only the public awareness of termly medical examination, but also a simple method to assess the kidney function. Due to the invasiveness, inconveniency and high cost, measuring GFR by the clearance of some exogenous markers is unsuitable in routine clinical practice, although they are the gold-standard methods [5]. Under such circumstance, estimating equations of GFR have gained booming development.

Among a large number of variations, the Modification of Diet in Renal Disease (MDRD), serum creatinine-based Chronic Kidney Disease Epidemiology Collaboration (hereafter referred to as CKD-EPI_2009Scr_) and MacIsaac equations have been publicly approved and applied [6]. Ma et al. developed the Chinese adapted MDRD equation (hereafter referred to as C-MDRD), which was validated to be better than other MDRD equations in Chinese subjects [7]. However, in patients with near-normal kidney function, the MDRD equations underestimate GFR [8]. The CKD-EPI_2009Scr_ equation partly overcomes the major limitation of the MDRD equation [9]. The MacIsaac equation is a typical cystatin C-based equation developed in 2006. The investigators challenged the traditional view that cystatin C level was independent of body composition. They proved that accounting for body composition improved cystatin C-based GFR estimation [10]. Some researchers shifted their focus to equations based on combination of different markers, for example, the combination of serum creatinine and cystatin C [11]. Ma equation is a representative, which is also based on the data from the Chinese population. They found the equation performed better than the C-MDRD equation, especially in early detection of CKD [12]. Our previous work evaluated the performance of existing equations, showing that they have their own applicability in different CKD stages and age groups [13].

Recently, the CKD-EPI working group has reported two new CKD-EPI equations: one using cystatin C concentration (CKD-EPI_2012cys_) and the other using both cystatin C and serum creatinine concentrations (CKD-EPI_2012Scr-cys_). They validate the new equations represent an advance over currently available equations across the range of GFR and in relevant subgroups. The advance even holds true among participants with an extreme body-mass index (the weight in kilograms divided by the square of the height in meters) of less than twenty [14]. The two new equations even have been recommended by KDIGO 2012Clinical Practice Guidelines for the Evaluation and Management of CKD [15]. A series of research for validation of the new formulae has appeared. Mindikoglu et al. evaluated the performance of CKD-EPI_2012Scr-cys_ equation in subjects with cirrhosis, claiming that it was superior to conventional equations for estimating GFR; however, the diagnostic performance was not as good as reported in non-cirrhotic subjects [16]. Obiols et al. found CKD-EPI_2012Scr-cys_ equation was more accurate and precise in hypertensive patients with higher GFR [17]. Kilbride et al. tested the accuracy of the new equations in old people in London [18]. Few data were available in China about the comparison of new equations with other traditional formulae. We compared the adaptability of new formulae with other four equations.

## Materials and Methods

### Participants

Totally 788 Chinese participants older than 18 years, with or without CKD at the First Affiliated Hospital of Nanjing Medical University between December 2009 and March 2012, were consecutively enrolled in the study. All participants in this study signed the informed consent. The ethics committee of Nanjing Medical University approved the study.

The participants with severe heart failure, acute renal failure, pleural or abdominal effusion, serious edema or malnutrition, skeletal muscle atrophy, amputation, ketoacidosis were excluded. Patients who were taking trimethoprim or cimetidine or ACEI/ARB and those who had recently received glucocorticoid and hemodialysis therapy were also excluded.

### Measurement and Estimation of GFR

Patients were informed in advance to avoid any meat consumption on the day of the test. Demographic data and past history were recorded and blood pressure, weight, and height were documented. Serum creatinine (Scr) concentration was assayed by the enzymatic method (Shanghai Kehua Dongling Diagnostic Products Co., Ltd, China), traceable to National Institute of Standards and Technology creatinine standard reference material (SRM 967).Cystatin C concentration was examined by the particle-enhanced immunoturbidimetry method (Beijing Leadman Biomedical Co., Ltd, China),which was calibrated against the international certified reference material ERM-DA471. Both fasting serum samples were assayed on an Olympus AU5400 autoanalyser (Olympus Co., Japan), in strict accordance with the manufacturer's instructions.

All participants had a ^99^mTc-DTPA renal dynamic imaging measurement as the reference glomerular filtration rate (rGFR), who had been required to have no special change in diet. After measuring height and weight, drinking300 ml water, and emptying the bladder, participants received a bolus injection in the elbow vein of 185 MBq 99mTc-DTPA (purity 95%–99%, Nanjing Senke Co., Ltd, China). The ^99^mTc-DTPA renal dynamic imaging measurement was carried out and after images acquisition, rGFR was automatically calculated with a computer by the Gates method.

Estimated glomerular filtration rate (eGFR) was calculated separately from six GFR estimating equations including the C-MDRD, MacIsaac equation, Ma equation, CKD-EPI_2009Scr_, CKD-EPI_2012cys_ and CKD-EPI_2012Scr-cys_ equations. The results are presented in detail in Table 1.

**Table 1:** Equations to predict glomerular filtration rate.

Name	Year	Gender	Scr	Scys	Equation
C-MDRD	2006				175×Scr^−1.234^×age^−0.179^(×0.79,if female)
MacIsaac	2006				(86.7/Scys)-4.2
Ma	2007				169×Scr^−0.608^×Scys^−0.63^×age^−0.157^(×0.83,if female)
CKD-EPI_2009Scr_	2009	female	≤0.7		144× (Scr/0.7)^−0.329^×0.993^age^(×1.159,if black)
			>0.7		144× (Scr/0.7)^−1.209^×0.993^age^(×1.159,if black)
		male	≤0.9		141× (Scr/0.9)^−0.411^×0.993^age^(×1.159,if black)
			>0.9		141× (Scr/0.9)^−1.209^×0.993^age^(×1.159,if black)
CKD-EPI_2012cys_	2012	female		≤0.8	133× (Scys/0.8)^−0.499^×0.996^age^×0.932
				>0.8	133× (Scys/0.8)^−1.328^×0.996^age^×0.932
		male		≤0.8	133× (Scys/0.8)^−0.499^×0.996^age^
				>0.8	133× (Scys/0.8)^−1.328^×0.996^age^
CKD-EPI_2012Scr-cys_	2012	female	≤0.7	≤0.8	130× (Scr/0.7)^−0.248^× (Scys/0.8)^−0.375^×0.995^age^(×1.08,if black)
				>0.8	130× (Scr/0.7)^−0.248^× (Scys/0.8)^−0.711^×0.995^age^(×1.08,if black)
			>0.7	≤0.8	130× (Scr/0.7)^−0.601^× (Scys/0.8)^−0.375^×0.995^age^(×1.08,if black)
				>0.8	130× (Scr/0.7)^−0.601^× (Scys/0.8)^−0.711^×0.995^age^(×1.08,if black)
		male	≤0.9	≤0.8	135× (Scr/0.9)^−0.207^× (Scys/0.8)^−0.375^×0.995^age^(×1.08,if black)
				>0.8	135× (Scr/0.9)^−0.207^× (Scys/0.8)^−0.711^×0.995^age^(×1.08,if black)
			>0.9	≤0.8	135× (Scr/0.9)^−0.601^× (Scys/0.8)^−0.375^×0.995^age^(×1.08,if black)
				>0.8	135× (Scr/0.9)^−0.601^× (Scys/0.8)^−0.711^×0.995^age^(×1.08,if black)

Note: Scr was shown as mg/dL; Scys was shown as mg/L; age was shown as years.

Abbreviations: Scr: serum creatinine; Scys: serum cystatin C; C-MDRD: the Chinese modified Modification of Diet in Renal Disease equation; CKD-EPI: Chronic Kidney Disease Epidemiology Collaboration; CKD-EPI_2009Scr_: serum creatinine–based CKD-EPI equation which was developed in 2009; CKD-EPI_2012cys_: cystatin C–based CKD-EPI equation which was newly developed in 2012; CKD-EPI_2012Scr-cys_: serum creatinine– and cystatin C–based CKD-EPI equation which was newly developed in 2012.

### Statistical Analysis

No data sets were normally distributed (P<0.001, Kolmogorov-Smirnov test); thus, nonparametric statistics were used throughout. The receiver operating characteristic (ROC) curve was depicted to analyse the diagnostic value of 6 equations. Abscissa of the curve is the value of (1- specificity) and the vertical is sensitivity. The larger area under the ROC curve (ROC^AUC^) usually means a better diagnostic value. Bias, precision and accuracy were used to evaluate the performance of each equation. Bias was defined as the median results of differences between eGFR and rGFR (eGFR-rGFR). The interquartile range (IQR) of the differences was a marker of precision. Accuracy was calculated as the proportion of eGFR within 30% of rGFR (P_30_) and also as root mean square error (RMSE). Wilcoxon matched-pairs signed rank test was used to compare the bias of each eGFR against rGFR. McNemar test was used to compare P_30_ values of the C-MDRD, MacIsaac, Ma, CKD-EPI_2012cys_ and CKD-EPI_2012Scr-cys_ equations against the P_30_ value of the CKD-EPI_2009Scr_ equation.

Bias plots were used to compare eGFR with rGFR intuitively. The difference between eGFR and rGFR was regressed against the mean of rGFR and eGFR. The greater slope of regression line against the x-axis means the larger bias. The larger intercept of the regression line against the y-axis indicates poorer accuracy. Kappa test was used to compare the diagnosis consistency of GFR stage between the eGFR and mGFR: kappa value 0.21–0.40 is considered mild agreement, 0.41–0.60 moderate agreement, 0.61–0.80 substantial agreement and 0.81–1.00 near-perfect agreement. Data was considered statistically significant at p<0.05. All statistical analyses were performed using SPSS software (version 17.0; SPSS, Chicago, IL, USA), Epical software (version 1.01; EpiCalc 2000 Application, Brixton Books, USA) and Medcalc for Windows (version 11.6.1.0; Medcalc Software, Mariekerke, Belgium).

## Results

### Participant characteristics

A total of 788 participants (478 male and 310 female), with or without CKD were enrolled. Median rGFR, cystatin C and Scr were 76.35 (interquartile range, 59.03–92.50) mL/min/1.73 m^2^, 1.09 (interquartile range, 0.92–1.43) mg/L and 0.94(interquartile range, 0.74–1.22) mg/dL, respectively. Participants were divided into two groups with rGFR<60 and ≥60 mL/min/1.73 m^2^ and also divided into two groups with age <60 and ≥60 years old. The detailed laboratory and anthropometric measurements are shown in Table 2.

**Table 2:** Characteristics of the Study Population.

	All subjects	Age<60	Age≥60
Age(years)	54(41–65)	45(34–53)	69(64–75)
Gender			
Male	478(60.70)	298(59.60)	180(62.5)
Female	310(39.30)	202(40.40)	108(37.5)
Weight(kg)	65(55–69)	65(55–68)	65(56–70)
Height(m)	1.69(1.60–1.70)	1.70(1.60–1.70)	1.68(1.60–1.70)
BSA(m^2^)	1.74(1.57–1.79)	1.73(1.56–1.78)	1.75(1.60–1.79)
BMI(kg/m^2^)	22.49(21.48–24.49)	22.49(21.48–24.22)	22.49(21.48–24.89)
BUN(mmol/L)	5.75(4.52–7.54)	5.33(4.17–6.78)	6.74(5.29–9.22)
Scr(mg/L)	0.94(0.74–1.22)	0.85(0.68–1.06)	1.09(0.88–1.51)
Scys(mg/L)	1.09(0.92–1.43)	0.98(0.84–1.20)	1.34(1.10–1.93)
rGFR(mL/min/1.73 m^2^)	76.35(59.03–92.50)	85.35(70.83–100.45)	62.85(46.35–74.85)
GFR category			
≥60 mL/min/1.73 m^2^	584(74.11)	425(85.00)	159(55.21)
<60 mL/min/1.73 m^2^	204(28.59)	75(15.00)	129(44.79)
eGFR(mL/min/1.73 m^2^)			
C-MDRD	87.27(62.91–111.25)	99.39(75.78–123.62)	66.35(45.24–85.99)
MacIsaac	73.99(54.88–89.50)	82.58(66.33–96.75)	58.30(40.50–73.80)
Ma	84.82(58.92–107.25)	96.61(76.16–116.41)	63.31(41.15–80.31)
CKD-EPI_2009Scr_	83.57(58.90–102.16)	95.82(76.18–110.28)	61.22(41.19–78.68)
CKD-EPI_2012cys_	67.75(45.62–90.63)	81.71(61.19–99.22)	47.57(30.45–64.65)
CKD-EPI_2012Scr-cys_	75.80(51.41–94.67)	88.00(69.42–103.28)	54.06(34.45–70.38)
Comorbid conditions			
Nephritis	46(5.84)	28(5.60)	18(6.25)
Kidney neoplasm	221(28.04)	132(26.40)	89(30.90)
Hematological disease	111(14.09)	101(20.20)	10(3.47)
Hypertension	162(20.56)	70(14.00)	92(31.94)
Coronary heart disease	35(4.44)	7(1.40)	28(9.72)
Diabetic mellitus	84(10.66)	26(5.20)	58(20.14)

Note: Values for continuous variables expressed as median (inter-quartile range); values for categorical values expressed as number (percentage). Conversion factors for units: serum creatinine in mg/dL to µmol/L, ×88.4.

Abbreviations: BSA: body surface aera; BMI: body mass index; Scr: serum creatinine; Scys: serum cystatin C; rGFR: reference glomerular filtration rate (using the ^99^mTc-DTPA renal dynamic imaging method); eGFR: estimated glomerular filtration rate; C-MDRD: the Chinese modified Modification of Diet in Renal Disease equation; CKD-EPI: Chronic Kidney Disease Epidemiology Collaboration; CKD-EPI_2009Scr_: serum creatinine–based CKD-EPI equation which was developed in 2009; CKD-EPI_2012cys_: cystatin C–based CKD-EPI equation which was newly developed in 2012; CKD-EPI_2012Scr-cys_: serum creatinine– and cystatin C–based CKD-EPI equation which was newly developed in 2012.

### Performance of six equations in all participants

Compared with CKD-EPI_2009Scr_, CKD-EPI_2012Scr-cys_ equation had a larger ROC^AUC^ (0.879 vs. 0.845) and higher sensitivity (88.7% vs.77.0%) but lower specificity (87.2% vs.92.1%) to diagnose CKD. CKD-EPI_2012cys_ equation did not perform much better than other available equations, except for its sensitivity (Table 3).

**Table 3:** Diagnostic value of six estimating equations compared with rGFR.

All subjects	R	ROC^AUC^	sensitivity	specificity
C-MDRD	0.795(p_1_ = 0.9)	0.853(p_2_ = 0.4)	75.5	95.0
MacIsaac	0.789(p_1_ = 0.6)	0.866(p_2_ = 0.1)	84.8	88.4
Ma	0.828(p_1_ = 0.08)	0.860(p_2_ = 0.1)	78.9	93.0
CKD-EPI_2009Scr_	0.798	0.845	77.0	92.1
CKD-EPI_2012cys_	0.802(p_1_ = 0.8)	0.852(p_2_ = 0.7)	92.2	78.3
CKD-EPI_2012Scr-cys_	0.829(p_1_ = 0.07)	0.879(p_2_ = 0.006)	88.7	87.2

age<60 y	R	ROC^AUC^	sensitivity	specificity
C-MDRD	0.671(p_1_ = 0.8)	0.851(p_2_ = 0.05)	73.3	96.9
MacIsaac	0.673(p_1_ = 0.7)	0.862(p_2_ = 0.1)	78.7	93.6
Ma	0.720(p_1_ = 0.07)	0.851(p_2_ = 0.1)	73.3	96.9
CKD-EPI_2009Scr_	0.661	0.829	69.3	96.5
CKD-EPI_2012cys_	0.681(p_1_ = 0.6)	0.876(p_2_ = 0.06)	86.7	88.5
CKD-EPI_2012Scr-cys_	0.718(p_1_ = 0.09)	0.883(p_2_ = 0.005)	81.3	95.3

age≥60 y	R	ROC^AUC^	sensitivity	specificity
C-MDRD	0.804(p_1_ = 0.9)	0.833(p_2_ = 0.1)	76.7	89.9
MacIsaac	0.807(p_1_ = 0.9)	0.813(p_2_ = 0.9)	88.4	74.2
Ma	0.832(p_1_ = 0.4)	0.823(p_2_ = 0.3)	82.2	82.4
CKD-EPI_2009Scr_	0.806	0.809	81.4	80.5
CKD-EPI_2012cys_	0.807(p_1_ = 0.9)	0.731(p_2_ = 0.002)	95.3	50.9
CKD-EPI_2012Scr-cys_	0.834(p_1_ = 0.3)	0.792(p_2_ = 0.4)	93.0	65.4

Note: R =  coefficient of relationship between eGFR and rGFR; ROC^AUC^  =  aera under receiver operating characteristic curve.

Abbreviations: C-MDRD: the Chinese modified Modification of Diet in Renal Disease equation; CKD-EPI: Chronic Kidney Disease Epidemiology Collaboration; CKD-EPI_2009Scr_: serum creatinine–based CKD-EPI equation which was developed in 2009; CKD-EPI_2012cys_: cystatin C–based CKD-EPI equation which was newly developed in 2012; CKD-EPI_2012Scr-cys_: serum creatinine– and cystatin C–based CKD-EPI equation which was newly developed in 2012.

Correlation coefficients of the C-MDRD equation, MacIsaac equation, Ma equation, CKD-EPI_2012cys_ equation and CKD-EPI_2012Scr-cys_ equation were compared against that of CKD-EPI_2009Scr_ equation (p_1_).

ROC^AUC^ of the C-MDRD equation, MacIsaac equation, Ma equation, CKD-EPI_2012cys_ equation and CKD-EPI_2012Scr-cys_ equation were compared against that of CKD-EPI_2009Scr_ equation (p_2_).

**Figure 1:**
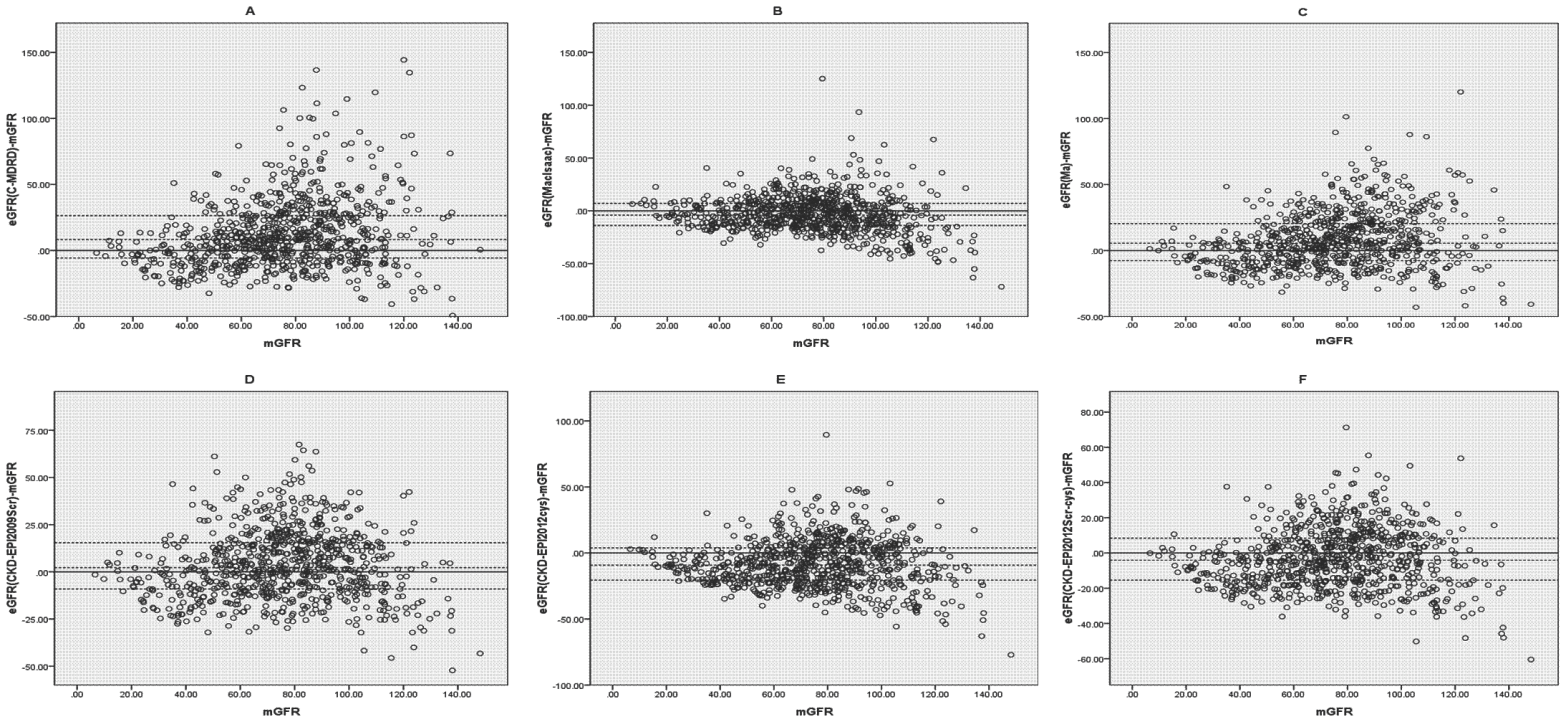
Bias plots intuitively compare estimated glomerular filtration rate (eGFR) with reference glomerular filtration rate (rGFR). The difference between eGFR and rGFR was regressed against the mean of rGFR and eGFR. The eGFRs were calculated separately from six estimating equations. (A) C-MDRD: the Chinese modified Modification of Diet in Renal Disease equation; (B) MacIsaac equation; (C) Ma equation; (D) CKD-EPI_2009Scr_: serum creatinine–based CKD-EPI equation which was developed in 2009; (E) CKD-EPI_2012cys_: cystatin C–based CKD-EPI equation which was newly developed in 2012; (F) CKD-EPI_2012Scr-cys_: serum creatinine– and cystatin C–based CKD-EPI equation which was newly developed in 2012. GFR were measured in mL/min/1.73 m^2^. Horizontal solid lines represent zero bias. Horizontal dashed lines represent 25th percentiles bias, median bias and 75th percentiles of the bias.

Performance of the equations is summarized in Table 4, and bias plots of the 6 equations against rGFR are shown in Figure 1. Both CKD-EPI_2012Scr-cys_ and CKD-EPI_2012cys_ underestimated GFR [(bias, −4.11; 95%CI, −5.17to−2.29 mL/min/1.73 m^2^; P<0.001) and (bias, −9.23; 95%CI, −10.60 to−7.40 mL/min/1.73 m^2^; P<0.001,) respectively] in the whole cohort. CKD-EPI_2009Scr_, Ma and C-MDRD equations overestimated GFR. Of all the 6 equations, CKD-EPI_2009Scr_ possessed the smallest difference (bias, 2.21; 95%CI, 0.73to4.58 mL/min/1.73 m^2^; P<0.001). MacIsaac, CKD-EPI_2012Scr-cys_ and CKD-EPI_2009Scr_ equation appeared to be more accurate with higher P_30_ value (80.20%, 77.03% and 75.76%, respectively).CKD-EPI_2012cys_ equation performed not as well as previously expected (P_30_ value, 68.40%). Concurrently, CKD-EPI_2012Scr-cys_ equation had the lowest RMSE and relative lower IQR.

**Table 4:** Performance of six estimating equations compared with rGFR.

All subjects	Bias (median difference) (95%CI)	Precision (IQR of the difference)	Accuracy P_30_ (95%CI)	Accuracy (RMSE)
C-MDRD	8.17(6.59,10.01)(p_3_<0.001)	32.11	64.21(60.74,67.55)(p_4_<0.001)	29.74
MacIsaac	−4.08(−5.58, −2.77)(p_3_<0.001)	20.88	80.20(77.21,82.90) (p_4_ = 0.02)	18.40
Ma	5.52(3.51,6.88)(p_3_<0.001)	28.01	71.19(67.87,74.31) (p_4_ = 0.003)	22.81
CKD-EPI_2009Scr_	2.21(0.73,4.58)(p_3_<0.001)	24.49	75.76(72.58,78.68)	18.94
CKD-EPI_2012cys_	−9.23(−10.60, −7.40)(p_3_<0.001)	24.39	68.40(65.01,71.61)(p_4_<0.001)	20.10
CKD-EPI_2012Scr-cys_	−4.11(−5.17, −2.29)(p_3_<0.001)	23.84	77.03(73.90,79.89)(p_4_ = 0.5)	17.29

rGFR≥60 mL/min/1.73 m^2^	Bias (median difference) (95%CI)	Precision (IQR of the difference)	Accuracy P_30_ (95%CI)	Accuracy (RMSE)
C-MDRD	11.54(9.33,14.10)(p_3_<0.001)	32.36	66.95(62.95,70.73) (p_4_<0.001)	32.84
MacIsaac	−4.22(−5.94, −2.41)(p_3_<0.001)	23.12	84.76(81.53,87.53)(p_4_ = 0.2)	20.06
Ma	8.80(6.71,10.79)(p_3_<0.001)	28.42	73.63(69.82,77.12)(p_4_<0.001)	25.07
CKD-EPI_2009Scr_	4.99(2.30,7.31)(p_3_<0.001)	25.67	81.68(78.25,84.68)	19.66
CKD-EPI_2012cys_	−7.37(−9.89, −4.41)(p_3_<0.001)	27.89	75.51(71.78,78.91)(p_4_ = 0.008)	21.30
CKD-EPI_2012Scr-cys_	−1.48(−3.82,1.13)(p_3_ = 0.09)	25.34	85.45(82.26,88.15)(p_4_ = 0.03)	18.07

rGFR<60 mL/min/1.73 m^2^	Bias (median difference) (95%CI)	Precision (IQR of the difference)	Accuracy P_30_ (95%CI)	Accuracy (RMSE)
C-MDRD	−0.22(−3.25,2.48)(p_3_ = 0.9)	23.10	56.37(49.27,63.23)(p_4_ = 0.4)	18.14
MacIsaac	−3.91(−5.81, −1.53)(p_3_<0.001)	17.01	67.16(60.19,73.46)(p_4_ = 0.05)	12.47
Ma	−3.08(−5.33,0.67)(p_3_ = 0.03)	19.05	64.22(57.18,70.71)(p_4_ = 0.1)	14.51
CKD-EPI_2009Scr_	−2.48(−5.45, −0.43)(p_3_ = 0.07)	19.97	58.82(51.72,65.59)	16.72
CKD-EPI_2012cys_	−10.86(−13.38, −9.82) (p_3_<0.001)	16.29	48.04(41.04,55.11)(p_4_ = 0.02)	16.20
CKD-EPI_2012Scr-cys_	−8.07(−11.34, −5.37)(p_3_<0.001)	17.34	52.94(45.86,59.91)(p_4_ = 0.2)	14.83

age<60 y	Bias (median difference) (95%CI)	Precision (IQR of the difference)	Accuracy P_30_ (95%CI)	Accuracy (RMSE)
C-MDRD	11.68(9.03,14.08)(p_3_<0.001)	35.06	62.00(57.57,66.24)(p_4_<0.001)	34.21
MacIsaac	−3.21(−5.58, −1.25)(p_3_<0.001)	22.12	80.60(76.80,83.92)(p_4_ = 0.05)	20.40
Ma	10.48(8.36,13.11)(p_3_<0.001)	30.42	68.20(63.89,72.23)(p_4_<0.001)	26.01
CKD-EPI_2009Scr_	7.53(5.34,9.57)(p_3_<0.001)	26.19	75.60(71.55,79.25)	21.01
CKD-EPI_2012cys_	−4.55(−7.40, −2.82)(p_3_<0.001)	24.91	75.40(71.34,79.07)(p_4_ = 0.1)	20.54
CKD-EPI_2012Scr-cys_	1.60(−0.78,3.22)(p_3_ = 0.6)	24.11	82.00(78.29,85.21)(p_4_ = 0.001)	18.19

age≥60 y	Bias (median difference) (95%CI)	Precision (IQR of the difference)	Accuracy P_30_ (95%CI)	Accuracy (RMSE)
C-MDRD	3.01(1.14,5.86)(p_3_<0.001)	23.96	68.06(62.28,73.34)(p_4_ = 0.001)	19.70
MacIsaac	−4.64(−6.91, −3.06)(p_3_<0.001)	19.42	79.51(74.29,83.93)(p_4_ = 0.3)	14.28
Ma	0.54(−3.44,2.29)(p_3_ = 0.9)	18.19	76.39(70.97,81.09)(p_4_ = 1.0)	15.81
CKD-EPI_2009Scr_	−2.66(−4.67, −0.85)(p_3_ = 0.007)	18.01	76.04(70.61,80.77)	14.66
CKD-EPI_2012cys_	−14.16(−17.31, −11.05)(p_3_<0.001)	19.48	56.25(50.30,62.03)(p_4_<0.001)	19.31
CKD-EPI_2012scr-cys_	−9.46(−11.39, −7.05)(p_3_<0.001)	16.46	68.40(62.64,73.66)(p_4_ = 0.008)	15.60

Note: Bias =  median difference between eGFR and rGFR; P_30_ = the proportion of eGFR within 30% of rGFR; RMSE =  root mean square error.

Abbreviations: rGFR: reference glomerular filtration rate; CI: confidence interval; IQR =  the inter-quartile range of difference; C-MDRD: the Chinese modified Modification of Diet in Renal Disease equation; CKD-EPI: Chronic Kidney Disease Epidemiology Collaboration; CKD-EPI_2009Scr_: serum creatinine–based CKD-EPI equation which was developed in 2009; CKD-EPI_2012cys_: cystatin C–based CKD-EPI equation which was newly developed in 2012; CKD-EPI_2012Scr-cys_: serum creatinine– and cystatin C–based CKD-EPI equation which was newly developed in 2012.

Wilcoxon matched-pairs signed rank test was used to compare the difference between the eGFR and rGFR (p_3_); McNemar test was used to compare the P_30_ of the C-MDRD equation, MacIsaac equation, Ma equation, CKD-EPI_2012cys_ equation and CKD-EPI_2012Scr-cys_ equation against the P_30_ of CKD-EPI_2009Scr_ equation (p_4_).

Linear regression analysis also demonstrated a consistent result (Table 5). The MacIsaac, CKD-EPI_2012Scr-cys_ and CKD-EPI_2009Scr_ equations showed better correlation, with lower slope and smaller intercept.

**Table 5:** Regression analysis of the difference between eGFR and rGFR against the average of eGFR and rGFR.

All subjects	Slope of regression line with the X-axias(95% CI)	Intercept of regression line with the Y-axis(95%CI)
C-MDRD	0.53(0.49,0.58)(p_5_<0.001)	−30.91(−35.06, −26.76)(p_6_<0.001)
MacIsaac	0.13(0.08,0.18)(p_5_ = 0.001)	−12.73(−16.61, −8.85) (p_6_ = 0.4)
Ma	0.39(0.35,0.44)(p_5_<0.001)	−23.28(−26.91, −19.66)(p_6_ = 0.002)
CKD-EPI_2009Scr_	0.25(0.20,0.29)	−15.07(−18.77, −11.38)
CKD-EPI_2012cys_	0.21(0.16,0.26)(p_5_ = 0.3)	−22.97(−26.55, −19.40)(p_6_ = 0.003)
CKD-EPI_2012Scr-cys_	0.22(0.17,0.26)(p_5_ = 0.4)	−18.73(−22.05, −15.41)(p_6_ = 0.1)

rGFR≥60 mL/min/1.73 m^2^	Slope of regression line with the X-axias(95% CI)	Intercept of regression line with the Y-axis(95%CI)
C-MDRD	0.80(0.72,0.87)(p_5_<0.001)	−59.31(−66.71, −51.92)(p_6_<0.001)
MacIsaac	0.30(0.21,0.39)(p_5_ = 0.8)	−28.70(−36.55, −20.84)(p_6_ = 0.3)
Ma	0.57(0.50,0.65)(p_5_<0.001)	−41.79(−48.82, −34.77)(p_6_<0.001)
CKD-EPI_2009Scr_	0.32(0.24,0.40)	−22.95(−30.43, −15.48)
CKD-EPI_2012cys_	0.38(0.30,0.47)(p_5_ = 0.3)	−38.98(−46.16, −31.79)(p_6_<0.001)
CKD-EPI_2012Scr-cys_	0.31(0.23,0.38)(p_5_ = 0.8)	−27.46(−34.30, −20.63) (p_6_ = 0.4)

rGFR<60 mL/min/1.73 m^2^	Slope of regression line with the X-axias(95% CI)	**Intercept of regression line with the Y-axis(95%CI)**
C-MDRD	0.73(0.63,0.84)(p_5_ = 0.4)	−30.73(−35.79, −25.66)(p_6_ = 0.73)
MacIsaac	0.35(0.24,0.47)(p_5_<0.001)	−17.71(−22.66, −12.76)(p_6_ = 0.001)
Ma	0.56(0.45,0.66)(p_5_ = 0.1)	−24.97(−29.71, −20.23)(p_6_ = 0.2)
CKD-EPI_2009Scr_	0.67(0.56,0.78)	−29.48(−34.38, −24.59)
CKD-EPI_2012cys_	0.32(0.20,0.43)(p_5_<0.001)	−22.99(−27.62, −18.36)(p_6_ = 0.06)
CKD-EPI_2012Scr-cys_	0.44(0.34,0.55)(p_5_ = 0.003)	−24.90(−29.40, −20.40)(p_6_ = 0.2)

age<60 y	Slope of regression line with the X-axias(95% CI)	Intercept of regression line with the Y-axis(95%CI)
C-MDRD	0.63(0.56,0.70)(p_5_<0.001)	−41.53(−48.23, −34.84)(p_6_<0.001)
MacIsaac	0.19(0.11,0.26) (p_5_ = 0.3)	−17.79(−24.23, −11.35) (p_6_ = 0.3)
Ma	0.44(0.38,0.50)(p_5_<0.001)	−27.38(−33.37, −21.39)(p_6_ = 0.002)
CKD-EPI_2009Scr_	0.24(0.17,0.30)	−13.48(−19.75, −7.22)
CKD-EPI_2012cys_	0.22(0.15,0.29) (p_5_ = 0.8)	−23.06(−29.07, −17.04)(p_6_ = 0.03)
CKD-EPI_2012Scr-cys_	0.21(0.14,0.27) (p_5_ = 0.6)	−16.96(−22.56, −11.36)(p_6_ = 0.4)

age≥60 y	Slope of regression line with the X-axias(95% CI)	Intercept of regression line with the Y-axis(95%CI)
C-MDRD	0.42(0.34,0.49)(p_5_<0.001)	−20.78(−25.81, −15.74)(p_6_ = 0.03)
MacIsaac	0.08(0.01,0.16)(p_5_ = 0.04)	−8.53(−13.30, −3.77)(p_6_ = 0.2)
Ma	0.30(0.23,0.37)(p_5_ = 0.04)	−17.17(−21.78, −12.56)(p_6_ = 0.2)
CKD-EPI_2009Scr_	0.19(0.12,0.26)	−13.18(−17.86, −8.50)
CKD-EPI_2012cys_	0.09(0.01,0.17)(p_5_ = 0.06)	−17.90(−22.46, −13.33)(p_6_ = 0.2)
CKD-EPI_2012Scr-cys_	0.13(0.06,0.20) (p_5_ = 0.2)	−15.62(−19.85, −11.39) (p_6_ = 0.4)

Note: The slope of the regression line against the X axis stands for the bias for eGFR; the trend of accuracy for eGFR was expressed as the intercept of the regression line against the Y-axis. The difference between eGFR and rGFR was regressed against the average of eGFR and rGFR. X-axis represented the average of eGFR and rGFR. Y-axis represented the difference between eGFR and rGFR.

Abbreviations: eGFR: estimated glomerular filtration rate; rGFR: reference glomerular filtration rate; CI: confidence interval; C-MDRD: the Chinese modified Modification of Diet in Renal Disease equation; CKD-EPI: Chronic Kidney Disease Epidemiology Collaboration; CKD-EPI_2009Scr_: serum creatinine–based CKD-EPI equation which was developed in 2009; CKD-EPI_2012cys_: cystatin C–based CKD-EPI equation which was newly developed in 2012; CKD-EPI_2012Scr-cys_: serum creatinine– and cystatin C–based CKD-EPI equation which was newly developed in 2012.

ANCOVA test was used to compare the slopes (p_5_) and intercepts (p_6_) of the regression line of the C-MDRD equation, MacIsaac equation, Ma equation, CKD-EPI_2012cys_ equation and CKD-EPI_2012Scr-cys_ equation against the slope and intercept of CKD-EPI_2009Scr_ equation.

In the comparison of the diagnosis consistency of GFR stage between the eGFR and mGFR, no equation achieved substantial agreement in this study. The CKD-EPI_2012Scr-cys_ had a relatively better diagnosis consistency with a kappa value of 0.513.However, CKD-EPI_2012cys_ equation did not perform well in the whole cohort (Table 6).

**Table 6:** Comparison of the diagnosis consistency of GFR stage between the eGFR and rGFR.

	rGFR≥90	rGFR 60–89	rGFR<60	sum	Kappa
*All subjects*					
**C-MDRD**					
eGFR≥90	**197(87.9)**	169(46.9)	7(3.4)	373	
eGFR60–89	27(12.1)	**162(45.0)**	43(21.1)	232	0.480
eGFR<60	0	29(8.1)	**154(75.5)**	183	
**MacIsaac**					
eGFR≥90	**131(58.5)**	63(17.5)	0	194	
eGFR60–89	90(40.2)	**232(64.4)**	31(15.2)	353	0.505
eGFR<60	3(1.3)	65(18.1)	**173(84.8)**	241	
**Ma**					
eGFR≥90	**192(85.7)**	151(41.9)	3(1.5)	346	
eGFR60–89	32(14.3)	**168(46.7)**	40(19.6)	240	0.494
eGFR<60	0	41(11.4)	**161(78.9)**	202	
**CKD-EPI_2009Scr_**					
eGFR≥90	**188(83.90)**	143(39.7)	6(2.9)	337	
eGFR60–89	36(16.1)	**171(47.5)**	41(20.1)	248	0.483
eGFR<60	0	46(12.8)	**157(77.0)**	203	
**CKD-EPI_2012cys_**					
eGFR≥90	**135(60.3)**	66(18.3)	0	201	
eGFR60–89	79(35.3)	**177(49.2)**	16(7.8)	272	0.451
eGFR<60	10(4.5)	117(32.5)	**188(92.2)**	315	
**CKD-EPI_2012Scr-cys_**					
eGFR≥90	**157(70.1)**	91(25.3)	0	248	
eGFR60–89	65(29.0)	**196(54.4)**	23(11.3)	284	0.513
eGFR<60	2(0.9)	73(20.3)	**181(88.7)**	256	
Sum	224	360	204	788	
*Age<60 y*					
**C-MDRD**					
eGFR≥90	**180(88.2)**	125(58.6)	4(5.3)	309	
eGFR60–89	24(11.8)	**83(37.6)**	16(21.3)	123	0.412
eGFR<60	0	13(5.9)	**55(73.3)**	68	
**MacIsaac**					
eGFR≥90	**126(61.8)**	50(22.6)	0	176	
eGFR60–89	75(36.8)	**147(66.5)**	16(21.3)	238	0.458
eGFR<60	3(1.5)	24(10.9)	**59(78.7)**	86	
**Ma**					
eGFR≥90	**178(87.3)**	120(54.3)	1(1.3)	299	
eGFR60–89	26(12.7)	**88(39.8)**	19(25.3)	133	0.421
eGFR<60	0	13(5.9)	**55(73.3)**	68	
**CKD-EPI_2009Scr_**					
eGFR≥90	**176(86.3)**	122(55.2)	4(5.3)	302	
eGFR60–89	28(13.7)	**84(38.0)**	19(25.3)	131	0.391
eGFR<60	0	15(6.8)	**52(69.3)**	67	
**CKD-EPI_2012cys_**					
eGFR≥90	**132(64.7)**	60(27.1)	0	192	
eGFR60–89	65(31.9)	**119(53.8)**	10(13.3)	194	0.423
eGFR<60	7(3.4)	42(19.0)	**65(86.7)**	114	
**CKD-EPI_2012Scr-cys_**					
eGFR≥90	**153(75.0)**	79(35.7)	0	232	
eGFR60–89	49(24.0)	**124(56.1)**	14(18.7)	187	0.478
eGFR<60	2(1.0)	18(8.1)	**61(81.3)**	81	
Sum	204	221	75	500	
*Age≥60 y*					
**C-MDRD**					
eGFR≥90	**17(85.0)**	44(31.7)	3(2.3)	64	
eGFR60–89	3(15.0)	**79(56.8)**	27(20.9)	109	0.482
eGFR<60	0	16(11.5)	**99(76.7)**	115	
**MacIsaac**					
eGFR≥90	**5(25.0)**	13(9.4)	0	18	
eGFR60–89	15(75.0)	**85(61.2)**	15(11.6)	115	0.481
eGFR<60	0	41(29.5)	**114(88.4)**	155	
**Ma**					
eGFR≥90	**14(70.0)**	31(22.3)	2(1.6)	47	
eGFR60–89	6(30.0)	**80(57.6)**	21(16.3)	107	0.492
eGFR<60	0	28(20.1)	**106(82.2)**	134	
**CKD-EPI_2009Scr_**					
eGFR≥90	**12(60.0)**	21(15.1)	2(1.6)	35	
eGFR60–89	8(40.0)	**87(62.6)**	22(17.1)	117	0.501
eGFR<60	0	31(22.3)	**105(81.4)**	136	
**CKD-EPI_2012cys_**					
eGFR≥90	**3(15.0)**	6(4.3)	0	9	
eGFR60–89	14(70.0)	**58(41.7)**	6(4.7)	78	0.349
eGFR<60	3(15.0)	75(54.0)	**123(95.3)**	201	
**CKD-EPI_2012Scr-cys_**					
eGFR≥90	**4(20.0)**	12(8.6)	0	16	
eGFR60–89	16(80.0)	**72(51.8)**	9(7.0)	97	0.431
eGFR<60	0	55(39.6)	**120(93.0)**	175	
Sum	20	139	129	288	

Note: eGFR and rGFR were given in mL/min/1.73 m^2^; bold font cells represent agreement; data were expressed as n (percentage).

Abbreviations: rGFR: reference glomerular filtration rate; eGFR: estimated glomerular filtration rate; C-MDRD: the Chinese modified Modification of Diet in Renal Disease equation; CKD-EPI: Chronic Kidney Disease Epidemiology Collaboration; CKD-EPI_2009Scr_: serum creatinine–based CKD-EPI equation which was developed in 2009; CKD-EPI_2012cys_: cystatin C–based CKD-EPI equation which was newly developed in 2012; CKD-EPI_2012Scr-cys_: serum creatinine– and cystatin C–based CKD-EPI equation which was newly developed in 2012.

### Performance of six equations in subgroups

In young participants, CKD-EPI_2012Scr-cys_ equation had a larger ROC^AUC^ thanCKD-EPI_2009Scr_ equation (0.883 vs. 0.829, p = 0.005) to diagnose CKD. CKD-EPI_2012cys_ equation did not perform much better than other available equations, especially in old participants. In subgroups with rGFR≥60 mL/min/1.73 m^2^ or the group with age<60 years old, CKD-EPI_2009Scr_, Ma and C-MDRD equations continued to overestimate GFR and CKD-EPI_2012Scr-cys_ equation was unbiased whereas all equations underestimated GFR when rGFR<60 mL/min/1.73 m^2^.Accuracy of the CKD-EPI_2012Scr-cys_ equation was superior (higher P_30_) to that of the CKD-EPI_2009Scr_ and MacIsaac equations at rGFR≥60 mL/min/1.73 m^2^ or age<60 years old. Neither of the two new CKD-EPI equation achieved an ideal P_30_ value under the condition of rGFR<60 mL/min/1.73 m^2^ or age ≥60 years old (Table 4).

Linear regression analysis demonstrated a similar result that MacIsaac, CKD-EPI_2012Scr-cys_ and CKD-EPI_2009Scr_ formulae performed better than other 3 equations in young participants with normal or mildly- injured GFR.

As for the diagnosis consistency, neither of the two new equations performed well in old participants and the CKD-EPI_2012cys_ equation in particular. The CKD-EPI_2009Scr_ equation had a comparatively better diagnosis consistency with a kappa value of 0.501under the condition of age ≥60 years old (Table 6).

## Discussion

This study compared the adaptability of new formulae with other four equations in788 participants. The principal finding of the present study was that CKD-EPI_2012Scr-cys_ formula had better diagnostic value and accuracy in the entire participants, particularly in young participants with normally or mildly- injured GFR. CKD-EPI_2012cys_ equation did not perform much better than other available equations. Concurrently, CKD-EPI_2012Scr-cys_ had a better diagnosis consistency of GFR stage between the eGFR and rGFR, especially in young participants. An important issue has to be explained. In the present study, the unbalanced subgroups (the number of participants in rGFR ≥60 ml/min/1.73 m^2^ and the number of participants in age <60 years old were much greater than the other two subgroups) might result in selective bias.

There were some other findings in this study. The MacIsaac equation, another well-behaved formula, also possessed a good diagnostic value and an impressive accuracy, even in old participants with moderately-severely injured GFR. Meanwhile, it had a fine diagnosis consistency of GFR stage between the eGFR and rGFR. It is a typical cystatin C-based equation.

It is well-known that serum creatinine is a classic kidney function indicator; however, it is easily influenced by many factors, such as body mass, dietary intake, aging, and analytic problems with assay methods [19]. Consequently the serum creatinine-based equations have these inherent limitations. Cystatin C is an endogenous 13 kDa protein that is freely filtered at the glomerulus, and then almost completely reabsorbed and catabolized by proximal tubular epithelial cells with only small amounts excreted in the urine. Cystatin C generation was felt to be constant, which resulted in cystatin C-based equations having a tendency to replace creatinine-based equations for a time [20]–[25]. However, previous studies have found non-GFR determinants of cystatin C, including non-renal elimination, differences in generation among individuals, relation to such factors as inflammation, steroid use and thyroid disease [26]–[28]. Thus, we cannot simply say cystatin C and cystatin C-based equations are better than serum creatinine and its formulae. Recently, Rule et al. reported serum creatinine-based equations are better than cystatin C-based ones for evaluating risk factors associated with CKD [29]. Therefore, there is no perfect formula and clinicians should choose an appropriate one depending on different study objectives.

The CKD-EPI_2012Scr-cys_equation, using the combination of serum creatinine and cystatin C, provides more precise GFR estimates. The CKD-EPI working group explained that errors due to the non-GFR determinants of serum creatinine and cystatin C are independent and smaller in an equation that uses both markers than in an equation that uses only one marker. They also claimed the addition of race as a variable had improved the performance of the CKD-EPI_2012Scr-cys_equation [14]. Bouvet et al. revealed that estimation of GFR using the four covariates (cystatin C, serum creatinine, body weight, and age) was less biased and more precise [11]. Thus, equations based on combination of different markers might become the final recommendation.

There are two strengths in this study. First, most of the studies, especially the two Chinese equations (the C-MDRD equation and Ma equation), were based on the patients with CKD [7], [10], [12].But eGFR always plays a role of screening the patients who might contract CKD, suggesting that those who use the equation may be healthy or with other disorders. Our study covered a proportion of subjects who might not suffer from CKD, which means we have evaluated the six famous equations in a more general population. Second, in our previous work, we investigated whether formulae possessed different diagnostic values between non-elderly and elderly subjects. Kilbride et al. tested the accuracy of the new equations in 74-year-olds or older in London [18]. We attempted to identify the value of the new equations in both non-elderly and elderly subjects.

There are also some limitations of this study. First of all, sample size, especially the number of participants with rGFR <30 ml/min/1.73 m^2^, is limited, and this is a single center study. Additionally, participants were all recruited from different departments of the hospital, which means rarely there was absolutely healthy population, and none of the data sets came from the population of patients with markedly reduced muscle mass or malnutrition. Thirdly, rGFR measurement by ^99^mTc-DTPA renal dynamic imaging method was still used in this study. It is, however, different from the renal clearance of Inulin used in the Mac study and ^125^I-iothalamate clearance in CKD-EPI equations. Therefore, the inconsistent rGFR may partially affect the true values. A nephrourology committee recommended double plasma clearance as the rGFR; however, before a unified rGFR can be carried out globally, this study may provide some exploratory information for clinicians and researchers. Moreover, the spectrum of disease is not uniform and the disease effect cannot be eliminated.

In summary, CKD-EPI_2012Scr-cys_ formula had better diagnostic value and accuracy in the whole cohort; however, its performance was substantially worse in old subjects with moderately-severely injured GFR. CKD-EPI_2012cys_ equation did not perform much better than other available equations. No magic formula has existed and every equation has its own characteristics. A large-scale study with many subjects and cooperating centers to develop a new formula for the general Chinese is necessary and urgent. Combination of different indicators should be recommended or more ideal endogenous indicators remain to be identified. Additionally, with the progress of medicine and the extension of human life, many countries have stepped into an aging society where elderly CKD population is rapidly expanding. We need to develop special formulae for this special population.
